# Masked polycythaemia vera is genetically intermediate between *JAK2*V617F mutated essential thrombocythaemia and overt polycythaemia vera

**DOI:** 10.1038/bcj.2016.70

**Published:** 2016-08-19

**Authors:** I S Tiong, D A Casolari, T Nguyen, M J M Van Velzen, K Ambler, R J D'Andrea, D M Ross

**Affiliations:** 1 Haematology Directorate, SA Pathology/Royal Adelaide Hospital, Adelaide, South Australia Australia; 2 School of Medicine, University of Adelaide, Adelaide, South Australia, Australia; 3 Centre for Cancer Biology, University of South Australia/SA Pathology, Adelaide, South Australia, Australia; 4 School of Medical Sciences, VU University Medical Center, Amsterdam, The Netherlands; 5 Genetics and Molecular Pathology Directorate, SA Pathology, Adelaide, South Australia, Australia

Polycythaemia vera (PV) can be defined as a myeloproliferative neoplasm (MPN) with erythrocytosis and *JAK2*V617F (or an equivalent marker of clonality). While these simple criteria are sufficient for most cases of overt PV, an estimated 10–15% of PV patients do not have an elevated haemoglobin concentration (Hb) and may mimic *JAK2*-positive essential thrombocythaemia (ET). This poses a problem for accurate diagnosis and may also have clinical significance, since patients with ‘masked' PV (mPV) might receive less intense treatment leading to shortened survival compared with overt PV.^1^

Several studies have focussed on alternative criteria for erythrocytosis to improve diagnostic accuracy.^2, 3^ These data have led to the adoption of haematocrit (Hct) thresholds and lower Hb thresholds in the recent revision of the World Health Organization (WHO) criteria.^4^ Both the British Committee for Standards in Haematology (BCSH) and the 2008 WHO incorporated minor criteria that enable a diagnosis of PV without a *JAK2* mutation: one of these is the presence of erythropoietin (EPO)-independent erythroid burst-forming units (BFU-E).^5, 6^ EPO-independent BFU-E are strongly associated with the presence of a *JAK2* mutation especially with the loss of heterozygosity (LOH) for *JAK2*.^7^ Copy-number-neutral LOH in PV occurs through uniparental disomy, which in the large majority of cases results in replacement of the normal *JAK2* allele by the mutated *JAK2* allele through mitotic recombination. Whereas EPO-independent BFU-E are found in some *JAK2*-positive ET cases, the finding of an expanded LOH clone (⩾20% of BFU-E) is unusual in ET.^8^ We hypothesized that patients with mPV would show a *JAK2* clonal structure more closely resembling overt PV, and that this might have diagnostic utility.

For the purposes of this study, we defined mPV as a *JAK2*-positive MPN with Hb below the 2008 WHO cutoff for PV and either Hb above the sex-specific normal range or Hb above the middle of the normal range despite iron deficiency or bone marrow (BM) panmyelosis. Patients with WHO-2008-defined PV (*n*=20) and *JAK2*-positive ET (*n*=12) were selected for comparison. All patients consented to tissue banking, and the use of archived samples was approved by the relevant ethics committee. BFU-E were grown from either peripheral blood (PB) or BM mononuclear cells, plated in methylcellulose supplemented with no or low-dose EPO (0.02 IU ml^−1^ recombinant erythropoietin-α, Janssen–Cilag). Where possible at least 30 individual BFU-E per patient were genotyped by single-nucleotide primer extension assay for *JAK2*V617F. A total of 2984 colonies (28–127 per patient) were genotyped.

The clinical and laboratory characteristics of the three cohorts are summarized in Table 1. Median age at diagnosis for ET, mPV and overt PV was 65.5, 63.0 and 62.5 years respectively. There was a stepwise increase in Hb and Hct, and a decrease in platelet counts from ET to mPV to overt PV, consistent with previous reports.^1, 3^ Significant differences were observed in serum ferritin and uric acid concentrations, and a similar trend was seen for leukocyte counts, lactate dehydrogenase activity and for PB *JAK2*V617F allele burden.

*JAK2* LOH was detected in 5 ET patients (42%, Figure 1), always in a minority (<10%) of BFU-E, concordant with a previous report.^8^ In overt PV patients *JAK2* LOH was identified in 19 patients (95%), with clonal expansion in 13 patients (65%). In the mPV cohort LOH was found in 12 patients (71%), with expansion of the LOH clone in only 2 patients (12%). Significant differences were found between the distribution of *JAK2* genotypes among the cohorts (goodness-of-fit test, *P*<0.001). *Post hoc* analysis, adjusted for multiple comparisons, confirmed significant differences between ET-mPV, ET-PV and mPV-PV (all *P* <0.001).

Spearman's correlation was used to estimate the strength of linear association between routine diagnostic parameters and the PB leukocyte *JAK2* allele burden or the proportion of BFU-E showing *JAK2* LOH. On one hand, *JAK2* allele burden showed a weak positive correlation with Hb (*r*=0.36), Hct (*r*=0.42) and leukocyte counts (*r*=0.40), and a weak negative correlation with platelet counts (*r*=−0.32). On the other hand, *JAK2* LOH in BFU-E showed a slightly stronger positive correlation with measures of erythrocytosis: Hb (*r*=0.45) and Hct (*r*=0.55). The proportion of BFU-E with *JAK2* LOH was strongly correlated with the PB leukocyte *JAK2* allele burden (*r*=0.74). It is worth noting that the common practice of equating LOH (or ‘homozygosity') with an allele burden of >50% gave a high positive predictive value (12/12, 100%), but a poor negative predictive value (12/35, 34%) for true LOH in BFU-E. Lastly, we explored whether the presence of an expanded LOH clone could be used as a molecular surrogate for erythrocytosis to improve diagnostic accuracy. No ET patient was incorrectly reclassified using a cutoff of ⩾20% BFU-E showing LOH, but only 2/17 mPV patients would have been identified as overt PV by this criterion.

We compared the erythrocytosis criteria of the BCSH and WHO 2016 revision. Ten mPV or PV patients did not have a BM biopsy and, for the purposes of this comparison, were assumed to have BM panmyelosis. Using the BCSH criteria (Hct >0.52 for males and >0.48 for females)^6^ 8/17 mPV patients (47%) and all overt PV patients were categorized as PV. Using the WHO 2016 criteria (Hb >165 g L^−1^ or Hct >0.49 for males, Hb >160 g L^−1^ or Hct >0.48 for females),^4^ 10/17 mPV patients (59%) were categorized as PV. This analysis suggests that the WHO 2016 criteria are more sensitive to mPV than were for the WHO 2008 criteria. None of the ET patients included in this study had BM panmyelosis, therefore preventing misclassification, but 3/12 patients (25%) met the WHO 2016 erythrocytosis criteria. Whether similar specificity is achieved in routine practice remains to be seen, since the assessment of BM histology is not always straightforward: 4% of PV cases were classified as normocellular and 18% of ET cases were hypercellular in a previous study of inter-observer reliability.^9^

One of our patients progressed from mPV to overt PV, and we studied BFU-E from both time points. This 45-year-old male presented initially with deep vein thrombosis (Hb 174 g L^−1^, Hct 0.53, EPO 11 IU ml^−1^, BM panmyelosis and PB *JAK2*V617F 27%), and was treated with venesection and anticoagulation. He was lost to follow up and returned 7 years later with overt PV (Hb 235 g L^−1^, Hct 0.73, EPO 5 IU ml^−1^ and PB *JAK2*V617F 76%) accompanied by symptomatic splenomegaly. The initial BFU-E assay showed *JAK2* LOH in 9.6% and heterozygosity in 90.4% of the colonies. In contrast, the subsequent analysis showed 72.3% LOH and 27.7% heterozygous colonies. A previous study involving 16 patients retested after intervals of 2–36 months found stable proportions of heterozygous and homozygous colonies.^8^ Our case illustrates that expansion of the *JAK2* LOH clone can occur over an interval of years and is associated with a more proliferative erythrocytosis phenotype.

The definition of mPV has varied between studies. Barbui *et al.*^2^ emphasized the importance of BM histology by including only those with BM panmyelosis and one other WHO 2008 minor criterion, but with Hb below the erythrocytosis threshold. Patients with iron deficiency were excluded. Alvarez-Larran *et al.*^3^ selected patients with an increased radionuclide red cell mass measurement, but with Hb below the WHO 2008 erythrocytosis threshold. Our selection criteria for mPV were less stringent, and included a high proportion of patients in whom iron deficiency may have limited the degree of erythrocytosis, a problem that was recognized decades ago by Iland *et al.*^10^ Arguably, iron replacement should not be given to such patients, which means that no definitive diagnosis can be reached using the WHO criteria. Consequently, we retained these patients in our study since they represent a cohort with an unmet diagnostic need.

Our findings re-emphasize the concept that PV and ET are not discrete entities but represent ends of a spectrum of *JAK2*-mutated MPN.^11^ We show that mPV is intermediate between ET and overt PV in both its clinical and mutational profile. *JAK2* LOH might help to improve diagnostic accuracy in selected cases, but the overall impact of this approach was less than that achieved by refining the criteria for erythrocytosis. BFU-E assays are not widely available, but similar information could be obtained using SNP-array on PB leukocytes, a method that is sufficiently sensitive to detect expanded LOH clones (⩾20%) and is available in most major centres.

While we have focussed primarily on *JAK2* LOH, an interesting observation is that 14/15 mPV patients without an expanded LOH clone had ⩾50% *JAK2*-heterozygous BFU-E and very few wildtype colonies, whereas only 4/12 ET patients showed a similar pattern. This argues that mPV patients are not simply overt PV patients with a phenotype modified by iron deficiency. It is likely that the phenotype, as well as the clonal structure of a *JAK2*-mutated MPN, is influenced by other factors. For example, suppression of normal haematopoiesis by *JAK2*-mutated cells has been reported to occur through paracrine secretion of TNF-α,^12^ and could contribute to clonal dominance without LOH. Possible genetic determinants of the PV phenotype include germline variants (for example, *HBS1L-MYB* intergenic polymorphism),^13^ additional somatic mutations (for example, *TET2* or *DNMT3A*),^14^ and the order in which *JAK2*V617F and other mutations are acquired.^15^

## Figures and Tables

**Figure 1:**
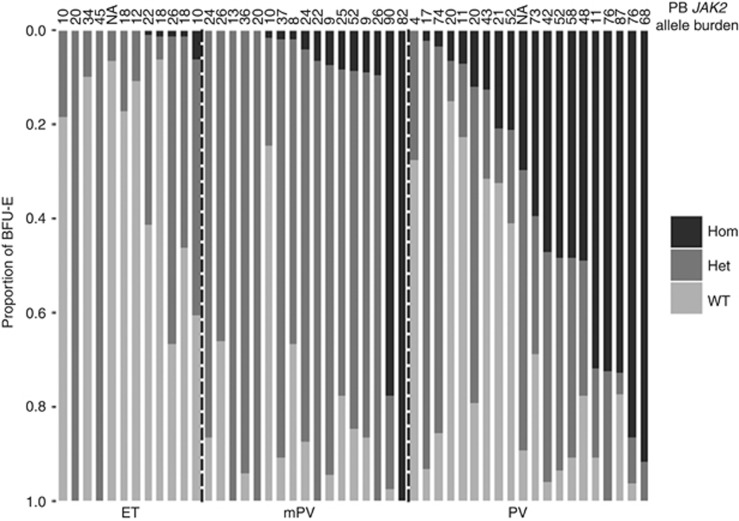
Distribution of *JAK2* genotypes in BFU-Es from patients with *JAK2*-mutated ET, mPV and overt PV. Each vertical bar represents a patient, divided according to the proportion of wild-type (WT), heterozygous (Het) and homozygous-mutant (Hom) colonies obtained. PB *JAK2*V617F allele burden is shown above each bar. The mean number of colonies genotyped per patient was: ET, 60.4; mPV, 51.7; and overt PV, 69.

**Table 1:** Characteristics of patients with JAK2-mutated ET, mPV and overt PV^a^

	*ET*	*Masked PV*	*Overt PV*
*N*	12	17	20
Age, years, median (range)	65.5 (44–86)	63.0 (34–86)	62.5 (40–88)
Male : Female	3:9	8:9	15:5

*Haemoglobin (g/L)*^b^			
Male	166.0^c^	175.5^c^	207.0^c^
	(160.6–169.6)	(164.1–183.6)	(187.0–240.7)
Female	143.0^c^	154.0^c^	175.0^c^
	(134.4–151.6)	(149.0–161.6)	(169.6–206.8)

*Haematocrit (%)*^b^			
Male	47.0^c^	53.0^c^	64.0^c^
	(45.2–49.7)	(51.4–58.0)	(57.0–73.3)
Female	43.0^c^	47.0	55.0^c^
	(40.8–47.4)	(44.4–51.2)	(50.8–64.8)
Leukocyte (x10^9^/L)	9.9	11.0	12.6
	(6.3–14.5)	(6.2–19.4)	(9.2–22.2)
Platelets (x10^9^/L)	696^c^	663^c^	432^c^
	(497-1359)	(197-1140)	(210-830)
EPO (IU/mL)^d^	6.5	4.0	4.0
	(6.0-6.9)	(2.0–10.0)	(1.0–9.0)
MCV (fL)	87.5	86.1	85.8
	(82.7–91.4)	(73.4–94.4)	(74.7–93.1)
MCV < 80 fL	0/12	5/17	5/20
Ferritin (μg/L)^e^	85.0^c^	49.0	24.5^c^
	(27.2-422.2)	(8.9–233.7)	(10.9–115.7)
Ferritin < 30 μg/L	1/9	6/14	12/20
PB leukocyte *JAK2* allele burden (%)^f^	18	24	46
	(10.0–39.5)	(8.8–83.6)	(10.3–77.1)
LDH (U/L)	249.0	272.0	308.5
	(141.8–508.8)	(164.8–443.0)	(203.3–484.2)
Urate (mmol/L)	0.31^c^	0.39	0.42^c^
	(0.24–0.47)	(0.24–0.54)	(0.28–0.59)
Number of BFU-E colonies (%)	725	879	1380
Homozygous mutant	7 (1.0)	110 (12.5)	445 (32.2)
Heterozygous mutant	276 (38.1)	644 (73.3)	475 (34.4)
Wild type	442 (61.0)	125 (14.2)	460 (33.3)

a Unless specified, data presented are median (5-95th percentile).

b The Hb and Hct values reported are the highest recorded at diagnosis or in the preceding 3 months. Patients with a sustained, unexplained Hb increase of ⩾20 g/L exceeding 170 g/L in men or 150 g/L in women were also considered as overt PV.

c indicates significant difference between the labelled groups with the same symbol, on Dunn's test for multiple comparisons after a significant Kruskal-Wallis test.

d EPO levels available for 3 ET, 12 mPV, and 19 overt PV patients.

e Ferritin levels available for 9 ET, 14 mPV and 20 PV patients.

f 1 ET and 1 PV patient had only qualitative JAK2V617F assay.
